# miR-21-5p renal expression is associated with fibrosis and renal survival in patients with IgA nephropathy

**DOI:** 10.1038/srep27209

**Published:** 2016-06-06

**Authors:** Marie-Flore Hennino, David Buob, Cynthia Van der Hauwaert, Viviane Gnemmi, Zacharie Jomaa, Nicolas Pottier, Grégoire Savary, Elodie Drumez, Christian Noël, Christelle Cauffiez, François Glowacki

**Affiliations:** 1 Univ. Lille, CHU Lille - Service de néphrologie, Institut Pasteur de Lille, EA 4483 - IMPECS - IMPact of Environmental ChemicalS on human health, F-59000 Lille, France; 2 Centre Hospitalier de Valenciennes - Service de néphrologie, médecine interne et vasculaire, F-59300 Valenciennes, France; 3 Sorbonne Universités, UPMC Paris 06, Inserm, AP-HP, Hôpital Tenon, Pathology Department, UMR S 1155, F-75020 Paris, France; 4 Univ. Lille, Inserm, CHU Lille, UMR-S 1172 - JPArc - Centre de Recherche Jean-Pierre AUBERT Neurosciences et Cancer, F-59000 Lille, France; 5 Centre Hospitalier de Cambrai - Service de néphrologie, F-59400 Cambrai, France; 6 Univ. Lille, CHU Lille, EA 2694 - Santé publique: épidémiologie et qualité des soins, F-59000 Lille, France; 7 Univ. Lille, Inserm, CHU Lille - Service de néphrologie, U995 - LIRIC - Lille Inflammation Research International Center, F-59000 Lille, France

## Abstract

IgA nephropathy (IgAN) is the most prevalent primary glomerulonephritis, whose prognosis is highly variable. Interstitial fibrosis is a strong independent prognosis factor. Among microRNA involved in renal fibrogenesis, only few have been investigated in IgAN. In the context of IgAN, we aimed to analyze the role of miR-21-5p, miR-214-3p and miR-199a-5p, three established “fibromiRs” involved in renal fibrosis. Fifty-six IgAN biopsy specimens were retrospectively scored according to Oxford classification. Renal expression of miR-21-5p, miR-214-3p and miR-199a-5p were significantly associated with T score (miR-21-5p T0 RQ median = 1.23, T1 RQ = 3.01, T2 RQ = 3.90; miR-214-5p T0 RQ = 1.39, T1 RQ = 2.20, T2 RQ = 2.48; miR-199a-5p T0 RQ = 0.76, T1 RQ = 1.41, T2 RQ = 1.87). miR-21-5p expression was associated with S score (S0 RQ median = 1.31, S1 RQ = 2.65), but not miR-214-3p nor miR-199a-5p. In our cohort, poor renal survival was associated with high blood pressure, proteinuria and elevated creatininemia, as well as T and S scores. Moreover, renal expression of miR-21-5p, miR-214-3p were associated with renal survival. In conclusion, miR-21-5p, miR-214-3p and miR-199a-5p are three “fibromiRs” involved in renal fibrosis in the course of IgAN and miR-21-5p and miR-214-3p are associated with renal survival.

IgA nephropathy (IgAN) is the most prevalent primary glomerulonephritis leading to end stage renal disease (ESRD) in 20% to 40% of patients^1^,^2^. IgAN is an immune complex-mediated proliferative glomerulonephritis characterized by IgA mesangial deposition. New pathogenesis approaches of primary IgAN rely on a multihit model in patients with particular genetic backgrounds^3^. Broadly speaking, increased production of hypogalactosylated IgA1^4^ elicits an autoimmune response characterized by the specific synthesis of anti-glycan antibodies^5^ followed by the formation and deposition of pathogenic immune IgA – IgG complexes in the glomerular mesangium^6^ resulting in inflammation and fibrosis^7^,^8^ characterized by substantial tissue remodeling and destruction of functional renal tissue^9^.

The histologic features of IgAN are diverse and include active lesions represented by mesangial hypercellularity, endocapillary and/or extra-capillary proliferation, as well as scarring lesions characterized by glomerular sclerosis and interstitial fibrosis^10^. The Oxford IgAN classification is a pathologic scoring system based on the pattern and the severity of both proliferative (mesangial and endocapillary hypercellularity) and scarring (segmental glomerular sclerosis and interstitial fibrosis) changes, and represents an established prognostic factor of IgAN^11^,^12^. In line with original and replicative studies, interstitial fibrosis is a relevant histological prognosis factor^11^,^12^.

MicroRNAs (miRNA) consist of about 22 nucleotides that imperfectly hybridize to 3′-UTR or other regions of messenger RNAs, typically leading to translation inhibition or transcript degradation. The large number of miRNAs, currently exceeding 2,000 in human (http://www.mirbase.org/), and their poor binding specificities lead to a huge number of potential miRNA-target gene interactions, which is thought to regulate up to 60% of human mRNAs^13^. Although miRNAs are involved in virtually all physio-pathological processes^14^, their role in IgAN pathogenesis is still in its infancy^15^. Here, based on our previous and ongoing studies^16^,^17^,^18^, we focused on three well-established “fibromiRs”: miR-21-5p, miR-199a-5p and miR-214-3p^19^. Several experimental models have indeed unraveled the implication of these miRNAs in various experimental models of tissue fibrosis^16^,^20^,^21^ as well as fibroproliferative diseases^19^. In particular, these miRNAs are consistently upregulated during fibrosis and represent prominent regulatory factors of TGF**β** signaling, a major biological pathway driving fibrosis^17^,^22^,^23^.

In this study, we evaluated for the first time the renal expression of three established “fibromiRs” (miR-21-5p, miR-199a-5p and miR-214-3p) in IgAN, as well as their association with Oxford classification, and renal survival.

## Results

### Patient characteristics

This study included 61 patients whose clinical and biological characteristics are shown in Table 1. Mean age was 36.3 ± 13.6 years. At diagnosis, 19 (31.1%) of the patients exhibited hypertension, 56 (91.8%) had hematuria, and 54 (88.5%) displayed significant proteinuria (≥0.3 g/24h) with five patients (8.2%) having nephrotic syndrome. Thirty-two patients (52.5%) developed a renal failure (estimated glomerular filtration rate (eGFR) <60 mL/min/1.73 m^2^) at diagnosis with a mean serum creatinine of 32.8 ± 21.9 mg/L.

At diagnosis, blockers of renin-angiotensin-aldosterone system were widely used (51 (83.6%)). Furthermore, six patients (9.8%) received steroids; these six patients displayed severe renal disease (mean serum creatinine: 43.2 ± 20.9 mg/L, mean eGFR 20 mL/min/1.73 m^2^) with high proteinuria (mean 4.9 ± 2.7 g/24 h), and their evolution was pejorative, as they all progressed to ESRD.

### Pathological Features

Renal biopsy specimens were analyzed in 56/61 (91.8%) cases. Histological sections contained an average of 16.5 ± 10.1 glomeruli, including 26.1% globally sclerotic glomeruli. Regarding interstitial fibrosis, 32 (57.2%), 12 (21.4%), and 12 (21.4%) biopsy specimens were scored T0 (no or mild fibrosis), T1 (moderate fibrosis), and T2 (severe fibrosis), respectively. Pathological characteristics concerning the M, E, and S items of the Oxford classification are shown in Table 1. Extra-capillary proliferation was noticed in 16 (28.6%) cases, involving 17.2% ± 12.3% of the glomeruli.

### Association of miR-21-5p, miR-199a-5p and miR-214-3p renal expression with MEST score

**Figure 1:**
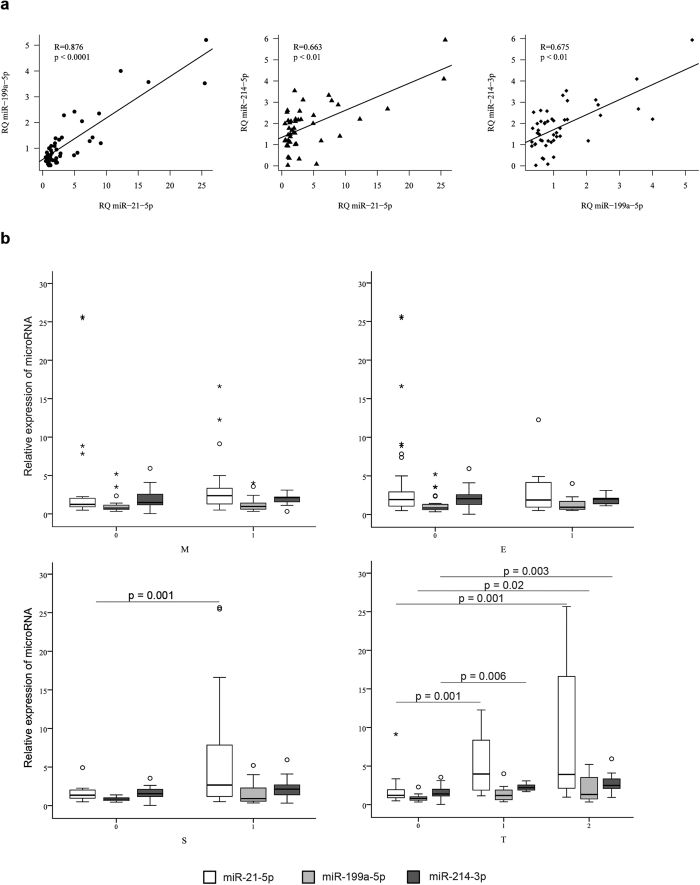
MiR-21-5p, miR-214-3p and miR-199a-5p are associated with segmental glomerulosclerosis (S) and interstitial fibrosis (T). (**a**) MiR-21-5p, miR-214-3p and miR-199a-5p renal expressions are correlated. (**b**) MiR-21-5p, miR-214-3p and miR-199-5p renal expression depending on the MEST score.

The renal levels of miR-21-5p, miR-199a-5p and miR-214-3p measured by RT-qPCR were highly correlated: R = 0.876, p < 0.0001 for miR-21-5p and miR-199a-5p, R = 0.663, p < 0.01 for miR-21-5p and miR-214-3p and R = 0.675, p < 0.01 for miR-199a-5p and miR-214-3p (Fig. 1a).

The expression levels of miR-21-5p, miR-199a-5p or miR-214-3p were not associated with the active glomerular lesions (*i.e.* mesangial hypercellularity (M) and endocapillary proliferation (E)) (Fig. 1b). By contrast, renal miR-21-5p, miR-199a-5p and miR-214-3p expression were significantly increased in patients with interstitial (T) and glomerular (S) fibrotic lesions. MiR-21-5p displayed the widest renal expression range amplitude (Fig. 1b). More precisely, miR-21-5p was found to be overexpressed in renal tissue of patients with moderate (T1: median RQ = 3.01 [1.13–12.26]) or severe (T2: median RQ = 3.90 [1.24–25.67]) fibrosis, compared with patients with mild fibrosis (T0: median RQ = 1.23 [0.48–9.12]). Similarly, miR-214-3p was overexpressed in renal tissue of patients with moderate (T1: median RQ = 2.20 [1.67–3.07]) or severe (T2: median RQ = 2.48 [2.03–5.93]) fibrosis, compared with mild fibrosis (T0: median RQ = 1.39[0.02–3.53]). Finally, compared to patients with mild fibrosis (T0: median RQ = 0.76 [0.33–2.27]), miR-199a-5p renal expression was significantly increased only in patients with severe fibrosis (T2: median RQ = 1.87 [0.61–5.20]) but not in patients with moderate fibrosis (T1: median RQ = 1.41). In addition, patients without glomerular sclerosis (S0), displayed lower miR-21-5p expression than patients with S1 lesions (p = 0.02) (S0: median RQ = 1.31 [0.48–2.25] *vs* S1: median RQ = 2.65 [0.50–25.67]), whereas expression levels of miR-199a-5p and miR-214-3p were not associated with glomerular segmental sclerosis (Fig. 1b).

Then to evaluate whether expression pattern of all three miRNAs could be used to predict disease severity, a principal component analysis (PCA) was performed. Active glomerular lesions were not associated to miRNAs expression pattern: (M0: median RQ = −0.63 [−1.66–6.59] *vs*. M1: median RQ = −0.24 [−1.19–3.09], p = 0.15 and E0: median RQ = −0.42 [−1.66–6.59] *vs*. E1: median RQ = −0.44 [−1.02–2.65], p = 0.74). Glomerular sclerotic lesions were also not associated to combined miRNAs (S0: median RQ = −0.59 [−1.66–0.73] *vs*. S1: median RQ = −0.21 [−1.35–6.59], p = 0.13). However, we confirmed a significant association with miRNAs expression and T score (T0: median RQ = −0.76 [−1.66–1.17] *vs*. T1: median RQ = 0.03 [−0.83–2.65] (p = 0.007) *vs*. T2: median RQ = 0.57 [−1.28–6.59] (p = 0.004)).

### Expression pattern of miR-21-5p in renal tissue

**Figure 2:**
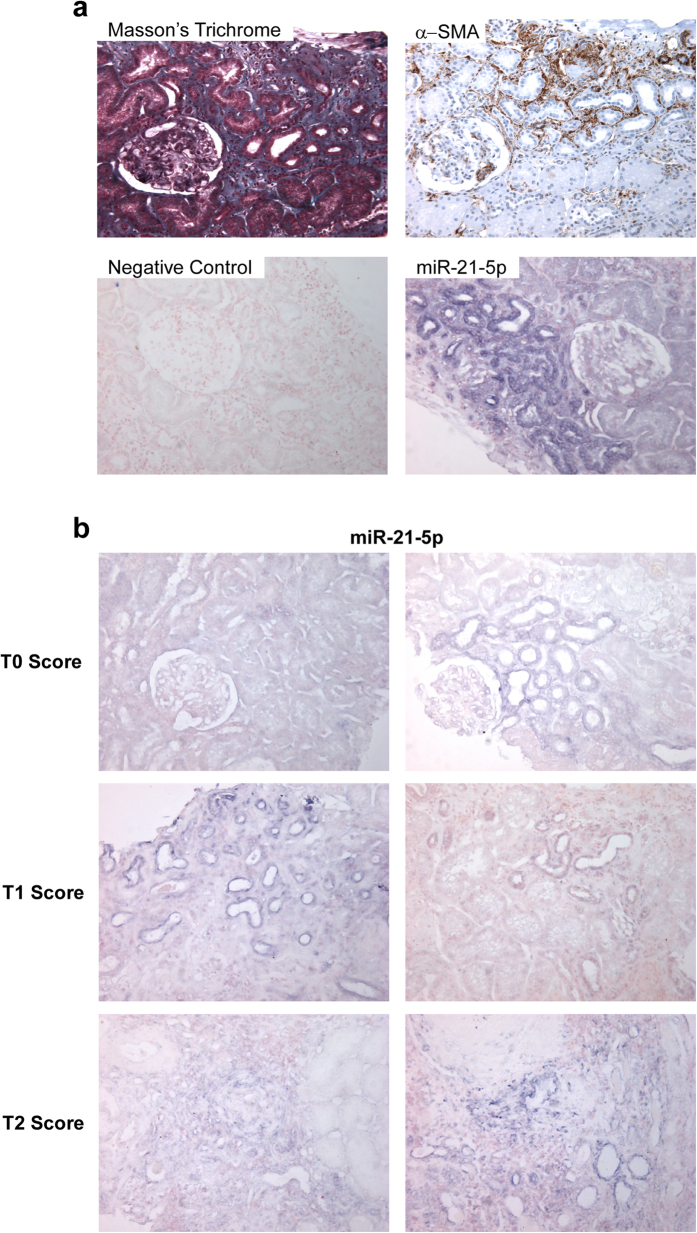
miR-21-5p is expressed in fibrotic areas. (**a**) miR-21-5p (*in situ* hybridization) is expressed in sub-atrophic tubules within fibrosis patches, highlighted by Masson’s trichrome coloration and α-SMA (α-smooth muscle actin) immunochemistry (200x). (**b**) *In situ* hybridization of miR-21-5p on biopsy according to fibrosis score (200x).

As miR-21-5p was strongly associated with fibrosis in IgA patients, tissue localization of miR-21-5p was evaluated by *in situ* hybridization on biopsy sections. miR-21-5p was primarily expressed in interstitial fibrotic areas, defined by both Masson’s trichrome and α-SMA (Smooth Muscle Actin) staining (Fig. 2a), and increased miR-21-5p expression was correlated with the severity of interstitial fibrosis (Fig. 2b). More precisely, miR-21-5p was strongly expressed in epithelial cells of subatrophic tubules, but not in normal epithelial cells. In biopsies with severe interstitial fibrotic lesions (T2), miR-21-5p expression was more prominent in areas associated with loss of normal tissue architecture.

### Determinants of renal survival

**Figure 3:**
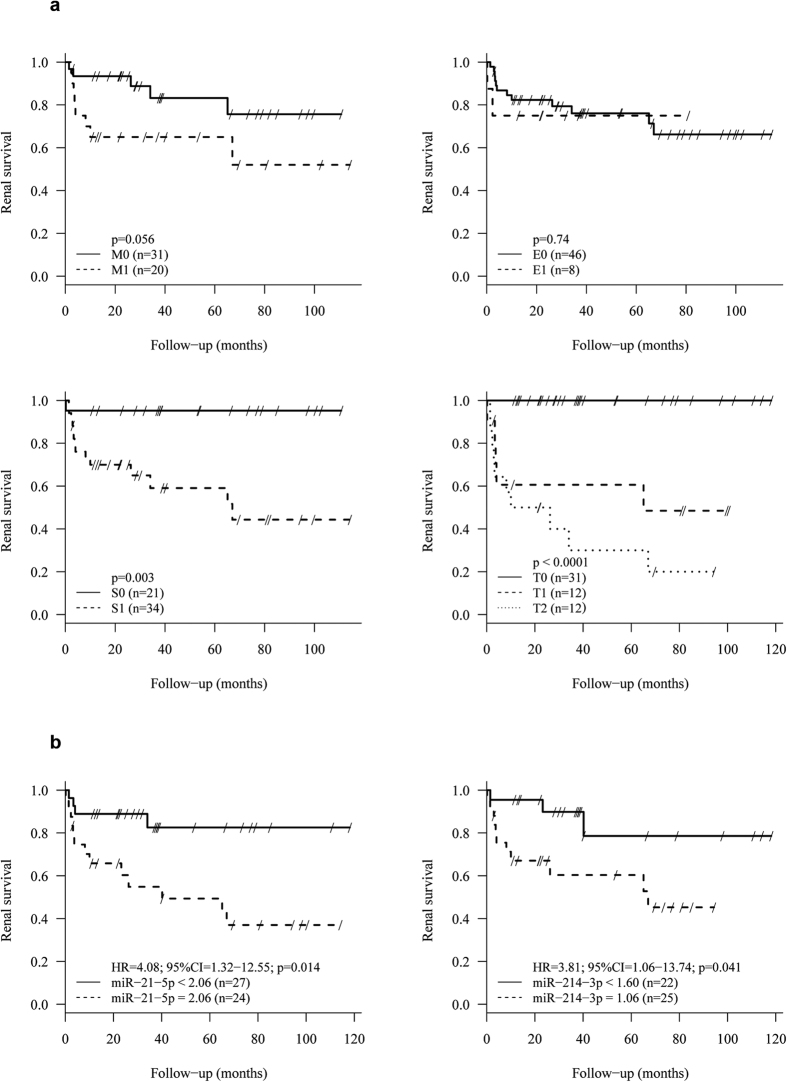
Renal survival is influenced by MEST score and renal expression of miRNAs in univariate analysis. (**a**) Renal survival is associated with M, S and T scores. (**b**) Renal expression of miR-21-5p and miR-214-3p are associated with renal survival.

During the follow-up (48.9 ± 38.1 months), 17 patients (27.9%) had an ESRD. Increased systolic blood pressure, proteinuria levels, decreased eGFR level, as well as T and S score were associated with renal failure (Table 2, Fig. 3a). Regardless of the renal expression levels of these miRNAs, a non-linear relationship between miRNA expression and the log relative risk of renal failure was found (data not shown). Using the optimal threshold values determined by maximization of hazard ratio, both miR-21-5p and miR-214-3p were significantly associated with risk of renal failure (Fig. 3b). Indeed, patients with miR-21-5p expression greater than 2.06 had significantly poorer renal survival (HR, 4.08; 95% CI, 1.32–12.55). Similar findings were found for patients with miR-214-3p expression greater than 1.60 (HR, 3.81; 95% CI, 1.06–13.74) (Table 2).

## Discussion

As IgAN outcome is highly variable and difficult to predict between individuals, the identification of prognostic factors at the time of presentation is of major interest, especially if these markers also represent targets for the development of anti-fibrotic therapeutics^7^. In this context, miRNAs represent attractive candidates as they have gained significant importance as diagnostic/prognostic markers, and constitute potentially innovative therapeutic targets. As developing miRNA antagonists is less challenging than miRNA mimics^19^,^24^, we chose in this study to focus our attention on three well-described pro-fibrotic miRNAs whose expression is consistently increased during fibrogenesis.

Although aberrant miRNA expression exerts a causative pathogenic role in most if not all complex diseases, their implication in IgAN is currently poorly documented^15^. To date, most studies have focused their efforts on the molecular mechanisms leading to the initiation of IgAN. For example, miR-148b and let-7b deregulation may be involved in the aberrant glycosylation of IgA1, which has a central pathogenic role in the early phase of IgAN^25^,^26^. However, understanding their role in the molecular events underlying kidney disease progression is of major interest since fibrosis has a very strong impact on IgAN outcome. In this context, Wang *et al*. reported that specific miRNA are upregulated (miR-141^27^ miR-205^27^, miR-192^27^, miR-146a^28^ and miR-155^28^) or downregulated (miR-200c^27^) in renal tissue of patients with IgAN compared with non inflammatory glomerulosclerosis. Moreover, renal miR-200c^27^, miR-146^28^ and miR-155^28^ and urinary miR-146a^28^, miR-155^28^, miR-200a^29^, miR-200b^29^, miR-429^29^, miR-29b^30^ and miR-29c^30^ are correlated with proteinuria while renal miR-205^27^, miR-192^27^, miR-146a^28^, miR-155^28^, and urinary miR-200b^29^, miR-429^29^, miR-21^30^, miR-29b^30^ and miR-29c^30^ are correlated with kidney function. The same team has also shown that renal expression of miR-205^27^ and miR-155^28^ are associated with interstitial fibrosis and renal miR-192^27^ and urinary miR-93^30^ are associated with glomerular sclerosis. Recently, Bao *et al*.^31^ showed that miR-21-5p was overexpressed in renal tissue of patients with IgAN compared with normal kidney tissue. Moreover, they positively correlated miR-21-5p glomerular levels with glomerular sclerosis and miR-21-5p tubulo-interstitial levels with interstitial fibrosis^31^. Here, we showed that renal levels of miR-21-5p, miR-199a-5p and miR-214-3p were significantly increased in IgAN patients exhibiting fibrotic changes. Compared to miR-199a-5p and miR-214-3p, miR-21-5p expression displayed greater and earlier variation. Moreover miR-21-5p was specifically associated with tubulointerstitial fibrosis and glomerular sclerosis but not with active proliferative glomerular lesions. Interestingly, patients with higher expression of miR-21-5p (>2.06) had a significantly worse renal survival. Clinico-biological and histological prognostic factors of IgAN in our cohort were in perfect agreement with the current prognosis factors included in the Oxford scoring system^11^,^12^. Indeed, high blood pressure, heavy proteinuria, and kidney failure are validated risk factors for developing ESRD in the context of IgAN. Similarly, interstitial fibrosis and glomerular sclerosis were two strong prognostic factors of renal survival. Furthermore, no association was found between endocapillary proliferation and renal survival. Taken together these data suggest that our cohort reflects the general population of patients with primary IgAN. Nonetheless, multivariate analysis was not possible given the limited number of patients and the high number of factors affecting renal survival. Therefore, our study should be interpreted as a pilot study and should be confirmed in larger cohorts.

Considering the data already observed about the role of miR-21-5p in other fibrosing kidney diseases such as chronic allograft dysfunction^16^ or diabetic nephropathy^32^, miR-21-5p is very likely a key player in renal fibrosis, regardless of the underlying renal disease. Although miR-21-5p dysregulation is not specific of IgAN, these results, particularly those concerning miR-21-5p, open new promising diagnostic and therapeutic options in the field of IgAN. Several studies have suggested the potential value of circulating miRNAs as diagnostic and/or prognostic non-invasive biomarkers^27^,^28^,^29^,^30^,^33^,^34^. Glowacki *et al*. have shown that the level of miR-21–5p in the serum of renal transplant recipients is associated with the severity of fibrosis^16^. Unfortunately, no sera or urine collection was available for our cohort of patients, and the predictive value of either blood or urinary miR-21-5p levels for renal scarring was not tested in the present study. Finally, miR-21-5p is a promising therapeutic target in renal fibrosis. Indeed, miR-21-5p knockout mice develop less renal fibrosis after unilateral ureteral obstruction compared to wild-type mice^21^. Moreover, administration of antagonist molecules of miR-21-5p successfully prevents the development of renal fibrosis in the unilateral ureteral obstruction^20^,^21^ as well as in the Alport syndrome^35^ mouse model. In this context, as no specific treatment is currently available for IgAN, targeted therapy against miR-21-5p may be an effective therapeutic option.

In conclusion, this work enabled to assess the involvement of three well-described pro-fibrotic miRNAs: miR-21-5p, miR-199a-5p and miR-214-3p, in the development of renal interstitial fibrosis occurring during IgAN. Among these candidates, miR-21-5p appears to be the most relevant. Its tissue expression significantly increases during development of fibrotic lesions in proportion to their severity and is also associated with renal survival. A prospective study is needed to confirm these data. Mechanistic studies to explore the regulatory pathways involving miR-21-5p, as well as IgAN mouse model will assess the relevance of a therapeutic strategy blocking miR-21-5p.

## Methods

### Ethics

This study was performed in accordance with the Declaration of Helsinki, with approval of the local ethic committee (CHU Lille). Renal biopsies were performed as described in our local regular clinical protocol. Informed consent was obtained for biopsy as well as for the use of clinical data and leftover histological material for research.

### Patients and clinical features

This was a single center retrospective study. A systematic review of the Pathology Department electronic database from 2002 to 2012 was performed to identify native kidney biopsy specimens with a histological diagnosis of IgAN. Patients with secondary forms of IgAN (Henoch-Schonlein purpura or cirrhosis) were excluded. Patients under renal replacement therapy (dialysis or kidney transplant) at the time of diagnosis were also excluded. Clinical (age, sex, weight, height, blood pressure) and biological (creatininemia, eGFR by MDRD formula, quantitative proteinuria, hematuria defined as >1+ by dipstick test) data at the time of biopsy were recorded. Treatments (renin angiotensin system blockade, corticosteroids, immunosuppressive agents), as well as evolution of clinical and biological data (at one year after diagnosis and at last follow-up time point) were collected. High blood pressure was defined as systolic blood pressure >140 mmHg, or diastolic blood pressure >90 mmHg, or previous antihypertensive drug prescription. Renal failure was defined as eGFR by MDRD <60 mL/min/1.73 m^2^. ESRD was defined as requirement for renal replacement therapy or transplantation.

### Histopathology

In the population of interest, the available biopsies were systemically reviewed by an experienced renal pathologist (D.B.) in a blind manner. Biopsies were scored according to the MEST criteria of the Oxford classification^10^,^11^. M score was derived from scoring each glomerulus according to mesangial hypercellularity (<4 cells per mesangial area = 0, 4–5 cells per mesangial area = 1, 6–7 cells per mesangial areas = 2, ≥8 cells per mesangial areas = 3). Biopsies were classified as M0 if the mean score of glomeruli was ≤0.5, or M1 if the mean score of glomeruli was >0.5. Endocapillary hypercellularity was classified E0 if absent and E1 if present. Segmental glomerulosclerosis was classified S0 if absent, and S1 if present. Interstitial fibrosis (score T) was estimated semi-quantitatively and classified as T0 if the fibrosis affected up to 25% of the cortical area, T1 between 26 and 50%, and T2 beyond 50%. We also recorded extra-capillary proliferation (cellular or fibro-cellular crescent). Vascular lesions were evaluated with arterial lesions (thickness of the intima <or> thickness of the media) and arteriolar lesions (proportion of vessels showing hyalinosis).

### Renal expression of miR-21-5p, miR-214-3p and miR-199a-5p

The extraction of total RNA, including miRNAs, was performed with four 10 μm-slices of paraffin embedded renal biopsies with the RecoverAll™ Total Nucleic Acid Isolation Kit (Thermofisher). Reverse transcription was performed using the TaqMan MicroRNA Reverse Transcription Kit (Thermofisher) with the specific TaqMan probe microRNA Assay (Thermofisher) for miR-21-5p (Assay ID397), miR-199a-5p (Assay ID498) and miR-214-3p (Assay ID517). U6-snRNA (Assay ID1973) was used as reference. Real-time PCR was performed with TaqManUniversal Master Mix II no UNG (Thermofisher) and miRNA specific primers (Thermofisher). The amplification reaction was performed on the device StepOne More Real Time PCR System (Thermofisher). Expression levels of mature miRNAs were calculated based on the comparative threshold cycle method (RQ = 2^−ΔΔCt^)^36^.

### *In situ* hybridization of miR-21-5p

Paraffin-embedded tissue sections (5 μm) were dewaxed with xylene and rehydrated in successive ethanol dilution. The sections were permeabilized by incubation in proteinase K (Thermofisher) for 15 minutes at 37 °C. After washing with PBS (Phosphate Buffer Salin) and dehydration, samples were incubated with the miR-21-5p Locked Nucleic Acid probe coupled to digoxigenin 50 nM (Exiqon) for 2 h at 45 °C. Slides were rinsed in 5X SSC (Sodium saline Citrate), 1X SSC and 0.2X SSC solutions at the same hybridization temperature and rinsed again with 0.2X SSC at room temperature The reaction was stopped with blocking solution (2% sheep serum, 2 mg/mL bovine serum albumine in PBS + 0.1% Tween 20) for 15 minutes at room temperature. Probe labeling was performed with sheep anti-DIG Fab fragments antibody conjugated to alkaline phosphatase (1/800) (Roche Applied Science). Revelation was carried out by incubation overnight at room temperature in a solution of 5-bromo-4-chloro-3-indyl phosphate and nitroblue tetrazolium (Roche Applied Science) with 0.2 mM levamisole. The color reaction was stopped by washing with 50 mM tris-HCl, 150 mM NaCl, 10 mM KCl in RNAse-free water. The sections were counterstained with Nuclear Fast Red to mark cell nuclei. Finally, slides were dehydrated and mounted with Eukitt^®^ (Dutscher).

### Immunostaining of α-SMA

On deparaffinized sections, the antigenic sites were unmasked with Tris buffer EDTA (ethylenediamine tetra-acetic acid) at pH 9 for 30 minutes. Immunostaining was performed using the PLC BenchmarkTX (Ventana) and XT ultra13 kit view diaminobenzidine (Ventana). The negative control was performed without the primary antibody.

Slides were incubated with PBS containing mouse monoclonal antibody anti-α-SMA diluted at 1/300 (Dako) at 4 °C overnight. The sections were then incubated with anti-mouse antibody conjugated with peroxidase-labeled polymer (Dako). Immunoreactive proteins were visualized with a 3-amino-9-ethylcarbazole-containing peroxidase substrate (hydrogen peroxide) (Dako). Finally, the tissue sections were counterstained with hematoxylin.

### Statistical analysis

Continuous variables are expressed as mean ± standard deviation or median [min-max]. Qualitative variables are expressed as count (percentage). Statistical significance between groups was assessed using ANOVA, unpaired Student’s t-test or non parametric test (Mann–Whitney or Kruskal–Wallis). Correlations between miRNAs renal expression were determined by calculating the Pearson correlation coefficient. Since miRNAs correlated with each other, principal component analysis (PCA) was performed to summarize the pattern of miR-21-5p, miR-199a-5p and miR-214-3p expression. The first principal component (PCA factor 1) who explained 83% of the variance was used as a measure of miRNA expression pattern in comparative analyses involving the MEST scores.

Renal failure-free survival, defined as ESRD requiring renal replacement, was estimated using the Kaplan-Meier method. We assessed the association of main biological and histological data, and the 3 microRNA with renal failure using the log-rank test for qualitative variables and univariable Cox proportional hazard regression models for continuous variables. For each continuous variable, the proportional hazards assumption was assessed by plotting the Schoenfeld residuals against the rank of survival time^37^ and the log-linearity assumption was assessed using Martingale residual plot and supremum test based on a sample of 1000 simulated residual patterns^38^. As the hypothesis of log-linearity was not reached for serum levels of miRNA, optimal threshold values were determined using the algorithm of maximization of hazard ratio^39^. In view of the study sample size, no multivariable analysis was performed.

Statistical testing was done at the two-tailed α level of 0.05. Statistical analyses were performed using the SPSS package, version 15.0 for Windows (Chicago, Illinois, USA) and SAS version 9.4 (SAS Institute, Cary, NC).

## Additional Information

**How to cite this article**: Hennino, M.-F. *et al*. miR-21-5p renal expression is associated with fibrosis and renal survival in patients with IgA nephropathy. *Sci. Rep.* **6**, 27209; doi: 10.1038/srep27209 (2016).

## Figures and Tables

**Table 1:** Clinical, biological and histological characteristics of the population. Values are means ± standard deviation.

*Clinical data(n *=* 61*)			
Age (years)	36.3 ± 13.6		
Sex ratio (men:women)	2.2:1		
Follow-up (months)	48.9 ± 38.1		
BMI (kg/m^2^)	25.7 ± 5.9		
HBP	19 (30.6%)		
- systolic BP (mmHg)	134 ± 21		
- diastolic BP (mmHg)	79 ± 16		
Prior RAAS blockade	24.2%		
***Biological data (n = 61)***			
Creatininemia (mg/L)	21.8 ± 19.6		
eGFR (mL/min/1.73 m^2^)	60 ± 36		
RF at diagnosis	37.7%		
Proteinuria (g/24h)	2.3 ± 2.3		
Nephrotic syndrom	5 (8.2%)		
Hematuria	56 (91.9%)		
***Histological data***(***n *****=***** 56***)			
M item	M0 n = 31 (55.4%)	M1 20 (35.7%)	NA 5 (8.9%)
E item	E0 46 (82.1%)	E1 8 (14.3%)	NA 2 (3.6%)
S item	S0 21 (37.5%)	S1 34 (60.7%)	NA 1 (1.8%)
T item	T0 32 (57.1%)	T1 12 (21.4%)	T2 12 (21.4%)
Extra-capillary proliferation	Absent n = 40 (71.4%)	Present n = 16 (28.6%)	

Abbreviations: BMI: Body Mass Index; HBP: High Blood Pressure, defined as systolic BP >140 mmH, or diastolic BP >90 mmHg, or previous antihypertensive drug intake; BP: Blood Pressure; RAAS: Renin-Angiotensin-Aldosteron System; eGFR: estimated Glomerular Filtration Rate by MDRD; RF: Renal Failure, defined as eGFR <60 mL/min/1.73 m^2^; NA: Not Available (no analyzable glomerulus).

**Table 2:** Univariate association of main clinico-biological characteristics and tissular microRNA with renal failure-free survival.

	Hazard Ratio	95% confidence interval	*p-V*alue
*Clinico-biological data*			
Systolic blood pressure*	1.59	1.29–1.97	<0.0001
Proteinuria^†^	1.57	1.30–1.89	<0.0001
eGFR^‡^	0.32	0.18–0.55	<0.0001
*microRNA*			
miR-21-5p ≥2.06	4.08	1.32–12.55	0.014
miR-214-3p ≥1.60	3.81	1.06–13.74	0.041
miR-199a-5p	NA		

eGFR: estimated Glomerular Filtration Rate, NA: Non Applicable. hazard ratio expressed per *10 mmHg increase of systolic blood pressure, ^†^1 g/day increase of proteinuria and ^‡^10 mL/min/1.73 m^2^ increase of eGFR.
